# R-SNARE Homolog MoSec22 Is Required for Conidiogenesis, Cell Wall Integrity, and Pathogenesis of *Magnaporthe oryzae*

**DOI:** 10.1371/journal.pone.0013193

**Published:** 2010-10-06

**Authors:** Wenwen Song, Xianying Dou, Zhongqiang Qi, Qi Wang, Xing Zhang, Haifeng Zhang, Min Guo, Suomeng Dong, Zhengguang Zhang, Ping Wang, Xiaobo Zheng

**Affiliations:** 1 Department of Plant Pathology, College of Plant Protection, Nanjing Agricultural University, and Key Laboratory of Monitoring and Management of Crop Diseases and Pest Insects, Ministry of Agriculture, Nanjing, China; 2 Department of Pediatrics, and the Research Institute for Children, Louisiana State University Health Sciences Center, New Orleans, Louisiana, United States of America; Louisiana State University, United States of America

## Abstract

Soluble N-ethylmaleimide-sensitive factor attachment protein receptor (SNARE) proteins mediate intracellular vesicle fusion, which is an essential cellular process of the eukaryotic cells. To investigate the role of SNARE proteins in the rice blast fungus *Magnaporthe oryzae*, MoSec22, an ortholog of *Saccharomyces cerevisiae* SNARE protein Sec22, was identified and the *MoSEC22* gene disrupted. MoSec22 restored a *S. cerevisiae sec22* mutant in resistance to cell wall perturbing agents, and the Δ*Mosec22* mutant also exhibited defects in mycelial growth, conidial production, and infection of the host plant. Treatment with oxidative stress inducers indicated a breach in cell wall integrity, and staining and quantification assays suggested abnormal chitin deposition on the lateral walls of hyphae of the Δ*Mosec22* mutant. Furthermore, hypersensitivity to the oxidative stress correlates with the reduced expression of the extracellular enzymes peroxidases and laccases. Our study thus provides new evidence on the conserved function of Sec22 among fungal organisms and indicates that MoSec22 has a role in maintaining cell wall integrity affecting the growth, morphogenesis, and virulence of *M. oryzae*.

## Introduction

Soluble N-ethylmaleimide-sensitive fusion protein attachment protein receptor (SNARE) proteins have been implicated as the conserved core protein machinery for intracellular membrane fusion events of eukaryotic cells including those of fungal organisms [1]. SNAREs are structurally characterised by a conserved segment of 60–70 amino acids, termed the SNARE motif, a cytoplasmically oriented N-terminal sequence, and a single transmembrane (TM) domain or lipid modification motif at the C-terminus [2], [3], [4]. Complementary SNAREs characterised by the “SNARE motif” are present on the donor and acceptor membranes prior to fusion, with conserved sequence features and a propensity to form coiled-coils configurations. When efficient member fusion occurs, four SNARE motifs bundle together forming a parallel α-helical coil. This transSNARE complex is referred to as a SNAREpin, and the formation of a SNAREpin pulls the vesicle and target membrane together and may provide the energy to drive fusion of the lipid bilayers [1], [5], [6], [7]. Functionally, SNAREs can be classified into v-SNAREs and t-SNAREs with the former associated with the vesicle and the latter the target compartment. Additionally, depending on sequence homology and the presence of an arginine or glutamine residue at the “0” layer of the SNARE motif, SNAREs can be categorised as R- or Qa-, Qb- and Qc-SNAREs [3].

SNAREs are a large family of proteins. There are 36 SNARE proteins found in humans, 20 in the fly *Drosophila melanogaster*, 54 in the plant *Arabidopsis thaliana*, and as many as 24 in the budding yeast *Saccharomyces cerevisiae* [8], [9], [10]. With the availability of many genome sequences, SNARE proteins were also identified from various filamentous fungi [11]. Kuratsu and colleagues identified 21 putative SNARE proteins from *Aspergillus oryzae*, and they also utilized the eGFP-markers attempting to localise these proteins [12]. Detailed knowledge on functions of SNARE proteins in these fungi remains lacking, and the only known example is Yup1 of the corn smut fungus *Ustilago maydis*. Yup1 was found to mediate endocytic recycling through early endosomes and is essential for hyphal morphogenesis and pathogenesis [13], [14].

The rice blast fungus *Magnaporthe oryzae* causes serious disease in a wide range of grass hosts, including rice, barley, wheat, and finger millet [15]. Additionally, *M. oryzae* was also widely regarded as a model fungus for studying the plant–microbe interaction [15], [16], [17]. Recently, molecular genetic analysis has led to the identification of secretomes containing a large and diverse set of secreted lytic proteins, such as xylanases, glucanases, cutinases, and additional plant cell wall-degrading enzymes that contribute to the pathogenicity of the fungus [18], [19]. Although a P-type ATPase (APT2) was shown to be essential for the release of several secretory proteins during the infection process [20], little is known about the mechanisms underlying the secretory transport of these lytic proteins to the periphery of the fungal cell walls.

The first step of secretion is the export of membrane and soluble cargos from the endoplasmic reticulum (ER) to the Golgi apparatus. In *M. oryzae*, two putative Hsp70 family proteins, Kar2 and Lhs1, were found to function as chaperones for protein translocation and maturation in the ER [21]. The *LHS1* gene is necessary for conidiation and for the ability to cause the rice blast disease, supporting a model that protein secretion is critical for development and pathogenesis. Studies in *S. cerevisiae* indicated that Sec22, one of the SNARE proteins, functions in both anterograde and retrograde traffic between the ER and the Golgi apparatus [22], [23]. The Δ*sec22* mutant exhibited defects in sporulation and budding, and the mutant cells were also less resistant to stress despite an increase in cell sizes [5], [24], [25].

Because SNARE family proteins are highly conserved among fungi, their studies may contribute to the understanding of secretory proteins and their role in pathogenesis of *M. oryzae*. We investigated the function of MoSec22, a *S. cerevisiae* Sec22 homologue, in *M. oryzae*. In the present study, we showed that MoSec22 was required for conidial development, stress resistance, and pathogenicity.

## Materials and Methods

### Fungal strains and growth condition

The *M. oryzae* wild-type Guy11 and mutant strains were cultured on complete medium (CM) (52) at 28°C. Other media include OMA (30 g oat meal and 15 g agar in 1 liter of distilled water) and V8 (100 ml V8 juice, 0.2 g CaCO_3_ and 15 g agar in 1 L ddH_2_O). For sporulation, RDC medium (100 g rice straw decoction was boiled in 1 L ddH_2_O for 20 min and filtered. The filtrate was mixed with 40 g corn meal and 10 g agar and adjusted to 1 L with ddH_2_O) was used [26]. For medium containing cell wall perturbing agents, the final concentrations were 0.01% for sodium dodecyl sulfate (SDS), 200 µg/ml for CR (Cong Red), and 200 µg/ml for Calcofluor White (CFW). Mycelia were harvested from 3-day-old cultures grown in liquid CM and used for genomic DNA and total RNA extractions.

### Protein sequence analysis

Sequence alignments were performed using the Clustal_W program [17] and the calculated phylogenetic tree was viewed using Mega4.0 Beta program [27].

### Complementation of the *Saccharomyces cerevisiae Δsec22 mutant*

*MoSEC22* was digested wi*th Eco*RI*-Hin*dIII from pMD-*MoSEC22*, and subcloned into the pYES2 yeast expression vector digested with *Eco*RI-*Hin*dIII to generate pYES2-*MoSEC22*, which expresses MoSec22 under the control of the *GAL1* promoter. After verification by sequencing, the pYES2-*MoSEC22* vector was introduced into the *S. cerevisiae* Δ*sec22* mutant strain YGL268w (BY4741: *Mata his3-1 leu2-0 met15-0 ura3-0 YGL268w::kanMX4*) purchased from EUROSCARF (Frankfurt, Germany) using the lithium-acetate method [28]. Yeast cells were incubated on liquid YPD medium (2% glucose, 2% peptone, and 1% yeast extract) supplemented with the amino acids required by the strains. After washed three times with ddH_2_O, aliquots (5 µl) of 10-fold serial dilutions were grown in SD (glucose) or SD-CFW (galactose+200 µg/ml CFW) plates at 30°C for 4 days and photographed.

### Construction of targeted gene deletion vector and fungal transformation

In order to analyze the effects of Δ*Mosec22* gene deletion, we created a construct of the targeted gene deletion vector pMD-*MoSEC22* KO by inserting the *HPH* gene expression cassette into the two flanking sequences of the *MoSEC22* gene. A 1.0 kb upstream flanking sequence fragment and a 0.8 kb downstream flanking sequence were amplified from *M. oryzae* genomic DNA by PCR, with primer pairs FL2450/FL2451 and FL2452/FL2453, respectively. Two PCR fragments were linked by overlap-PCR with primer pairs FL2450/FL2453, and the amplified products were cloned into pMD19-T vector (TaKaRa, Dalian, China) to generate pMD-*MoSEC22-*KO. An *Eco*RV restriction site was incorporated into primers FL2451/FL2452. The *HPH* gene cassette was prepared by PCR from the plasmid pCB1003 with primer pair FL1111/FL1112 and inserted into the *Eco*RV site of pMD-*MoSEC22* to generate the final construct pMD-*MoSEC22* KO. A 3.2 kb fragment containing the flanking sequence and *HPH* gene was amplified using the pMD-*MoSEC22* KO as the template with primers FL2450/FL2453 and used to transform *M. oryzae* strain Guy11 as described previously [29]. Primers were listed in Table S1.

Candidate mutant strains were screened using primers FL2580/FL2581 and the mutants were further verified by Southern blotting analysis and RT-PCR with primers FL2580/FL2581. For complementation, the 2.8-kb PCR product containing about 1.8 kb upstream sequence, the full-length *MoSEC22* gene coding region, and 0.4 kb downstream sequence were first obtained using primers FL3614/FL3615 and cloned into pCB1532 generating pCB1532-*MoSEC22R*. After sequence verification, the construct was used to transform the Δ*Mosec22* knockout mutants.

DNA and RNA manipulation as well as Southern blotting analysis were performed as described previously [30].

### RT-PCR and real-time PCR analysis

Total RNA was isolated using the RNA extraction kit MucleoSpin RNAII (MACHEREY-NAGEL, PA, USA). First-strand cDNA was synthesized using M-MLV Reverse Transcriptase (Invitrogen) and oligo(dT) 15 primers (Invitrogen). For semi quantitative RT-PCR, the *Actin* gene (MGG_03982.5) was amplified with primers FL474/FL475 and used as an internal control. The *MoSEC22* transcripts were obtained using primers FL2580/FL2581. In quantitative real-time PCR, *MgYAP1* (MGG_12814.6), *NOX1* (EF667340), and *NOX2* (EF667341) were amplified with primer pairs FL4348/FL2701, FL4394/FL4395, and FL4396/FL4397 respectively (Table S1). MGG_08200, MGG_07790, MGG_01313, MGG_01924, MGG_13239, MGG_04545, MGG_04404, MGG_13464, MGG_11608, MGG_09139, MGG_02069 were amplified using primer pairs FL4781/FL4782, FL4783/FL4784, FL4793/4794, FL4803/FL4804, FL4779/4780, FL4787/4788, FL4799/FL4800, FL4789/4790, FL4795/4796, FL4801/4802, FL4806/FL4807, respectively (Table S1). Quantitative real-time PCR was performed with the ABI 7300 Fast Real-Time System and transcripts were analyzed by the 7300 System SDS Software. To compare the relative abundance of target gene transcripts, the average threshold cycle (Ct) was normalized to that of *Actin* for each of the treated samples as 2^−ΔCt^, where −ΔCt  =  (C_t, target gene_ − C_t, actin_). Fold changes during fungal development and infectious growth compared to growth in liquid CM were calculated as 2^−ΔΔCt^, where −ΔΔCt  =  (C_t, target gene_− C_t, actin_) _test condition_ − (C_t, WT_ − C_t, actin_)_CM_ [31]. PCR was repeated once with three replicates.

### Assays for vegetative growth and appressorium formation in hyphal tips

Discs of mycelia 3 mm^2^ in size, from 7-day-old CM plates were individually incubated on the centre of 60-mm Petri dishes containing different media (CM, V8, OMA, and RDC) and cultured at 28°C with a 12 hours interval photophase. Radial growth was measured after incubation for 6 days. All experiments were repeated three times with three replicates each time.

To induce production of conidia, mycelia were allowed to grow on RDC medium at 28°C in the dark for 7 days, followed by constant illumination for 3–4 days [26]. Conidia were harvested by washing with ddH_2_O, filtered through three-layer lens paper, harvested by centrifugation, and cells resuspended in 0.2 ml ddH_2_O. Conidia concentration was counted using a hemocytometer. Three plates were used for each strain and the experiment was repeated three times. Conidiophore was monitored as previously described [32].

Appressorium formation was measured on GelBond film (FMC Bioproducts, Rockland, Maine, USA) as previously described, except that the conidial suspension was replaced with fragmented mycelia suspension. Mycelia fragments were suspended in sterile distilled water to ensure 10^4^ pieces of fragmented mycelia/ml. Droplets (40 µl) of the mycelia suspension were placed on GelBond film and incubated in a humid environment at 25°C. The percentage of appressoria formed at the tips of mycelia was determined microscopically. At least 100 pieces of fragmented mycelia per replicate were observed at 24 and 48 hours.

### Light microscopy studies

To examine hyphal morphology, strains were grown on a thin layer of CM agar on the microscope slides. After 2 days in a humid chamber at 28°C, the hyphae were observed under an Olympus BH-2 microscope. The cell wall, hyphal septum, and conidia were visualized by CFW (10 mg/ml, Sigma) staining as described [33].

### Pathogenicity assay

15-day-old rice (CO39) seedlings were grown under the conditions described previously [34]. Mycelia plugs of 2 mm×2 mm were removed from CM medium and incubated on the non-wounded or wounded rice leaves. Root infection assays were carried out as previously described [35]. Lesion formation was examined at 7 days post inoculation.

### Reaction oxygen species (ROS) detection

For superoxide detection, the strains were grown on a thin layer of CM on the microscope slides for 2 days in a humid chamber at 28°C. Hyphae were then stained with 0.3 mM NBT (nitroblue tetrazolium) aqueous solution for 20 min. The reaction was stopped by the addition of ethanol, and the pattern of formazan staining was observed using a Leica DMR microscope (Leica Microsystems, Wetzlar, Germany).

### Measurement of the chitin content

Chitin (N-acetylglucosamine, GlcNAc) content was determined as described [36]. Mycelial samples were freeze-dried first. For each sample, 5 mg of dried biomass was resuspended in 1 ml 6% KOH and heated at 80°C for 90 min. Samples were centrifuged (16,000 g, 10 min) and pellets washed with PBS in three cycles of centrifugation and resuspension (16,000 g, 10 min). The pellets were finally resuspended in 0.5 ml of McIlvaine's buffer (pH 6) with 100 µl (13 units) of *Streptomyces plicatus* chitinase (Sigma) and incubated for 16 hours at 37°C with gentle mixing. 100 µl sample was then combined with 100 µl of 0.27 M Mosadium borate (pH 9) in a 1.5 ml Eppendorf tube, heated for 10 min at 100°C, and 1 ml of freshly diluted (1∶10) of Ehrlich's reagent (10 g β-dimethylaminobenzaldehyde in 1.25 ml of concentrated HCl and 8.75 ml glacial acetic acid) was added. After incubating at 37°C for 20 min, 1 ml of the sample was transferred to a 2.5 ml plastic cuvette (Greiner) and the absorbance at 585 nm was recorded. Standard curves were prepared with GlcNAc (Sigma, USA). The experiment was repeated three times.

### Extracellular laccase activity and oxidative stress sensitivity assays

The laccase activity was monitored on 0.2 mM 2, 2′-azino-di-3-ethylbenzath- iazoline-6-sulfonate (ABTS) agar plate assays using mycelial plugs at 2 day post infection. The enzyme activity was also assayed using the culture filtrate from 3 day old CM liquid culture. Briefly, a reaction mixture (1 ml) containing 50 mM acetate buffer (pH 5.0) and 10 mM ABTS was mixed with the culture filtrate (200 µl) and incubated at 25°C for 5 minutes with or without 3 mM of H_2_O_2_. Absorbance was evaluated at 420 nm [37].

*M. oryzae* strains were grown on solid CM containing 2.5 and 5 mM H_2_O_2_ and the sensitivity was evaluated by measuring the colony diameters of 7-day-old cultures.

## Results

### Isolation of *M. oryzae SEC22* and functional complementation of a *S. cerevisiae* Δ*sec22* mutant

**Figure 1:**
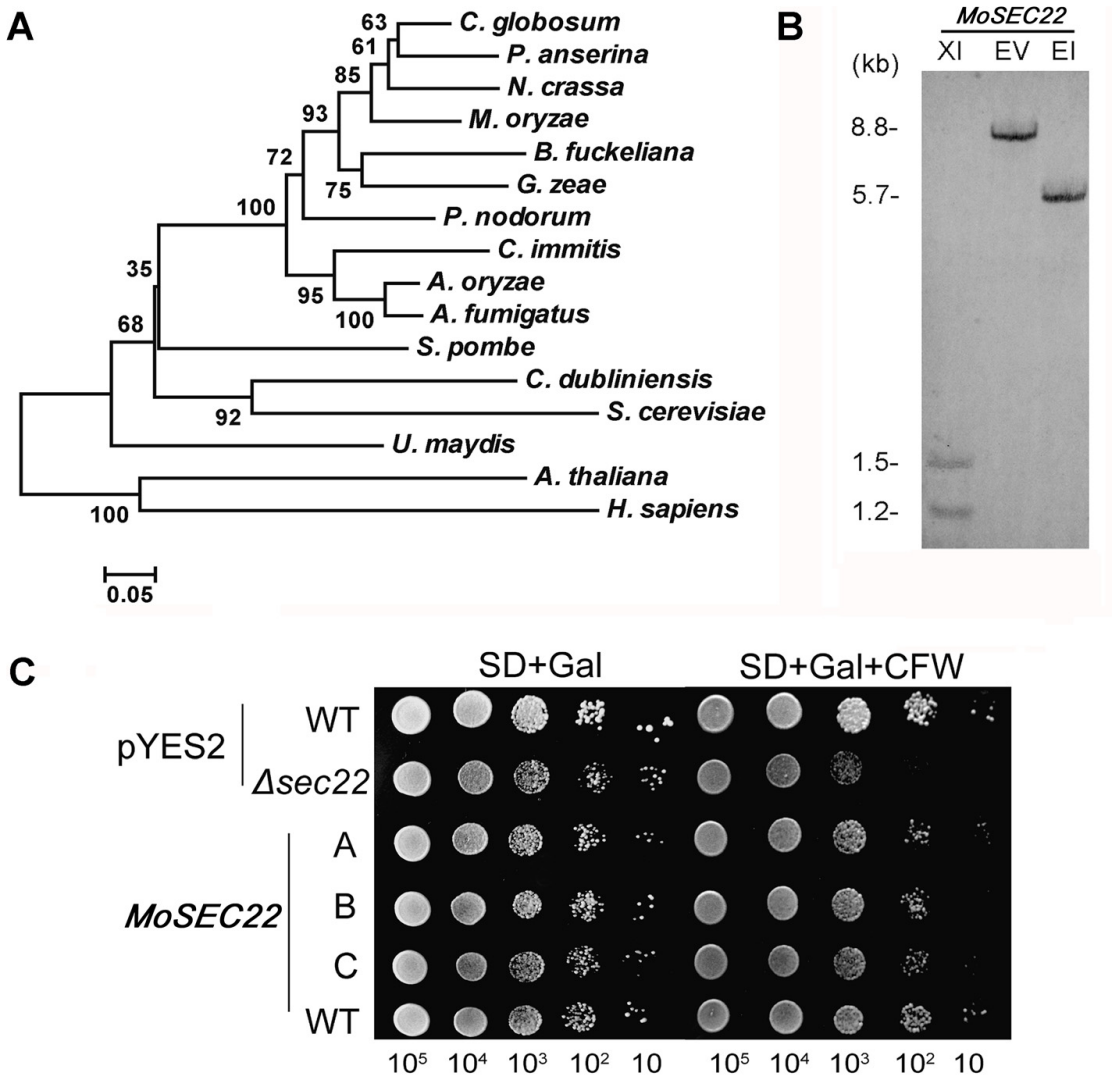
Phylogenetic tree of Sec22 from different organisms and targeted gene replacement and complementation of Δ*Mosec22.* (A) Phylogenetic tree of Sec22 proteins was constructed based on alignment of the full sequences of Sec22 from fungi to mammals: *Chaetomium globosum* (XP_001225428), *Podosporo anserine* (XP_001911329), *Neurospora crassa* (XP_960888), *Magnaporthe oryzae* (XP_361576), *Botryotinia fuckeliana* (XP_001553340), *Gibberella zeae* (XP_385402), *Phaeosphaeria nodorum* (XP_001792269), *Coccidioides immitis* (XP_001239175), *Aspergillus oryzae* (BAF36383), *Aspergillus fumigatus* (XP_747662), *Schizosaccharomyces pombe* (NP_596218), *Candida dubliniensis* (CAX39961), *Saccharomyces cerevisiae* (NP_013370), *Ustilago maydis* (XP_762021), *Arabidopsis thaliana* (NP_172653), and *Homo sapiens* (NP_004883). (B) Southern blotting analysis of *MoSEC22.* Genomic DNA of Guy11 strain was digested with *Xho*I (XI), *Eco*RV (EV) and *Eco*RI (EI), respectively, and separated in a 0.7% agarose gel. Numbers at left are molecular markers in kilobases. (C) The *MoSEC22* gene rescued the CFW sensitivity of the *S. cerevisiae* Δ*sec22* mutant. The *S. cerevisiae* Δ*sec22* mutant was transformed with the empty pYES2 vector and a pYES2-*MoSEC22* construct encoding MoSec22. The wild type was also transformed with the vectors. Serial dilutions of cultures of three independent transformants (A, B, and C) were grown overnight on SD-Met-Leu-His (glucose) or SG-Met-Leu-His (galactose +200 µg/ml CFW) plates, and then grown at 30°C for 4 days and photographed. Triangles represent decreases in the numbers of cells plated at each spots. The experiments were repeated at least three times with triple replications yielding similar results.

We first identified an ortholog of Sec22 from the *M. oryzae* genome (http://www.broad.mit.edu/annotation/genome/magnaporthe_grisea/MultiHome.html) by a BLAST_P search. *MoSEC22* (MGG_04050.6) encodes a polypeptide of 217 amino acids containing an intron-less SNARE domain. A high of 83% amino acid sequence identity was found to an uncharacterised hypothetical protein from *Chaetomium globosum* (CHGG07772), and the best match to an annotated Sec22 protein was that of *Neurospora crassa* (P78746; 79% identity in amino acid sequence). MoSec22 shares an overall of 47% in amino acid sequence with *S. cerevisiae* Sec22, while the identity within the SNARE domain (pfam00957) was as high as 60%. Further analysis of MoSec22 using the C-terminal SNARE motif revealed that the central position (0-layer) of the heptad repeats of the SNARE motif is an Arg residue, indicating that MoSec22 belongs to the R-SNARE superfamily. Alignment of Sec22 proteins was presented in Figure 1A. A single copy *MoSEC22* gene was found in *M. oryzae*, as indicated by Southern blotting analysis (Figure 1B).

MoSec22 was verified by functional complementation in *S. cerevisiae*. The *S. cerevisiae* Δ*sec22* mutant was highly sensitive to cell wall perturbing agent CFW [38]. To determine whether MoSec22 could complement Sec22 function, we expressed *MoSEC22* in a Δ*sec22* mutant through the yeast expression vector pYES2. The transformants exhibited better growth on synthetic defined medium containing 200 µg/ml CFW in comparison to the Δ*sec22* mutant carrying the empty vector (Figure 1C), suggesting that MoSec22 is able to fulfil the function of its counterpart in *S. cerevisiae*.

### Disruption of *MoSEC22* compromised aerial mycelial growth and inhibited conidiogenesis as well as appressorium formation

**Figure 2:**
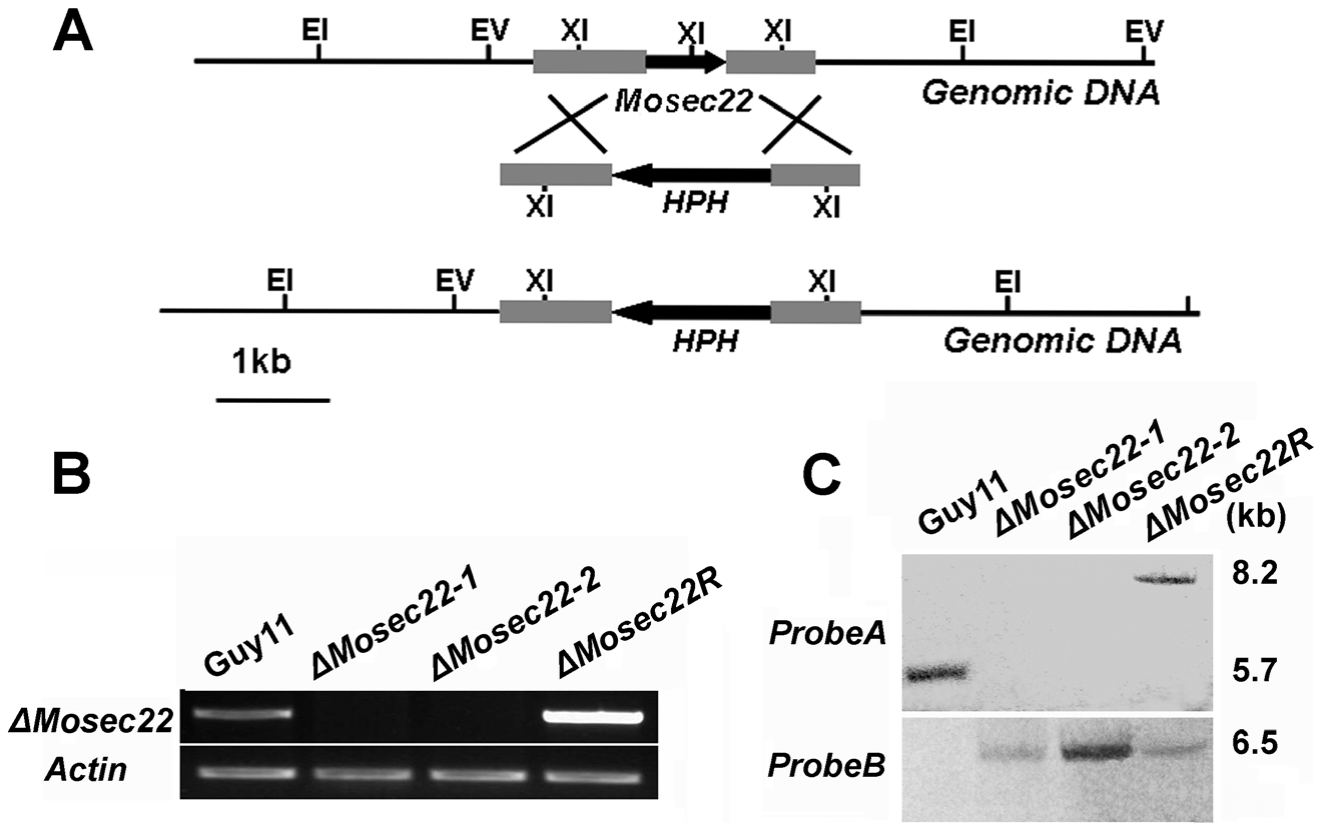
Targeted gene replacement and complementation of Δ*Mosec22*. (A) Illustration of the *MoSEC22* targeted gene replacement strategy. A 683 bp fragment of the *MoSEC22* coding region was replaced with a 1.4 kb fragment containing the *HPH* cassette to create a Δ*Mosec22* mutant allele, Scale bar  = 1 kb. (B) Semiquantitative RT-PCR was carried out to confirm the deletion and reintroduction of the *MoSEC22* gene. Data comprise three independent experiments with triple replications that yielded similar results. (C) Mutant transformants were verified by Southern blotting analysis. Genomic DNA was digested with *Eco*RI and separated in a 0.7% agarose gel. The DNA was hybridized with probe A, the 651 bp *MoSEC22* fragment and hybridized with a 5.7 kb fragment in wild type Guy11 and 8.2 kb fragment in Δ*Mosec22* for complementation. To validate the disruption of the *MoSEC22* gene, the genomic DNA of *MoSEC22* was digested with *Eco*RI, and hybridized with a 6.5 kb *HPH*-containing fragment.

To evaluate the role of MoSec22 in the growth and development of *M. oryzae*, disruption mutant strains were generated by replacing most of the *MoSEC22* coding region with the hygromycin phosphotransferase resistance (*HPH*) marker gene (Figure 2A). Mutant strains selected and conformed by Southern blot analysis and RT-CPR. Two deletion mutants, Δ*Mosec22-#1* and Δ*Mosec22-#2*, were selected after verified by Southern blot analysis and RT-PCR (Figure 2B and 2C). For complementation, a 2853-bp fragment containing the *MoSEC22* gene was ligated into pCB1532 vector [39] and reintroduced into the Δ*Mosec22*-#2 mutant. One strain, Δ*Mosec22R* was chosen once verified (Figure 2B and 2C).

**Figure 3:**
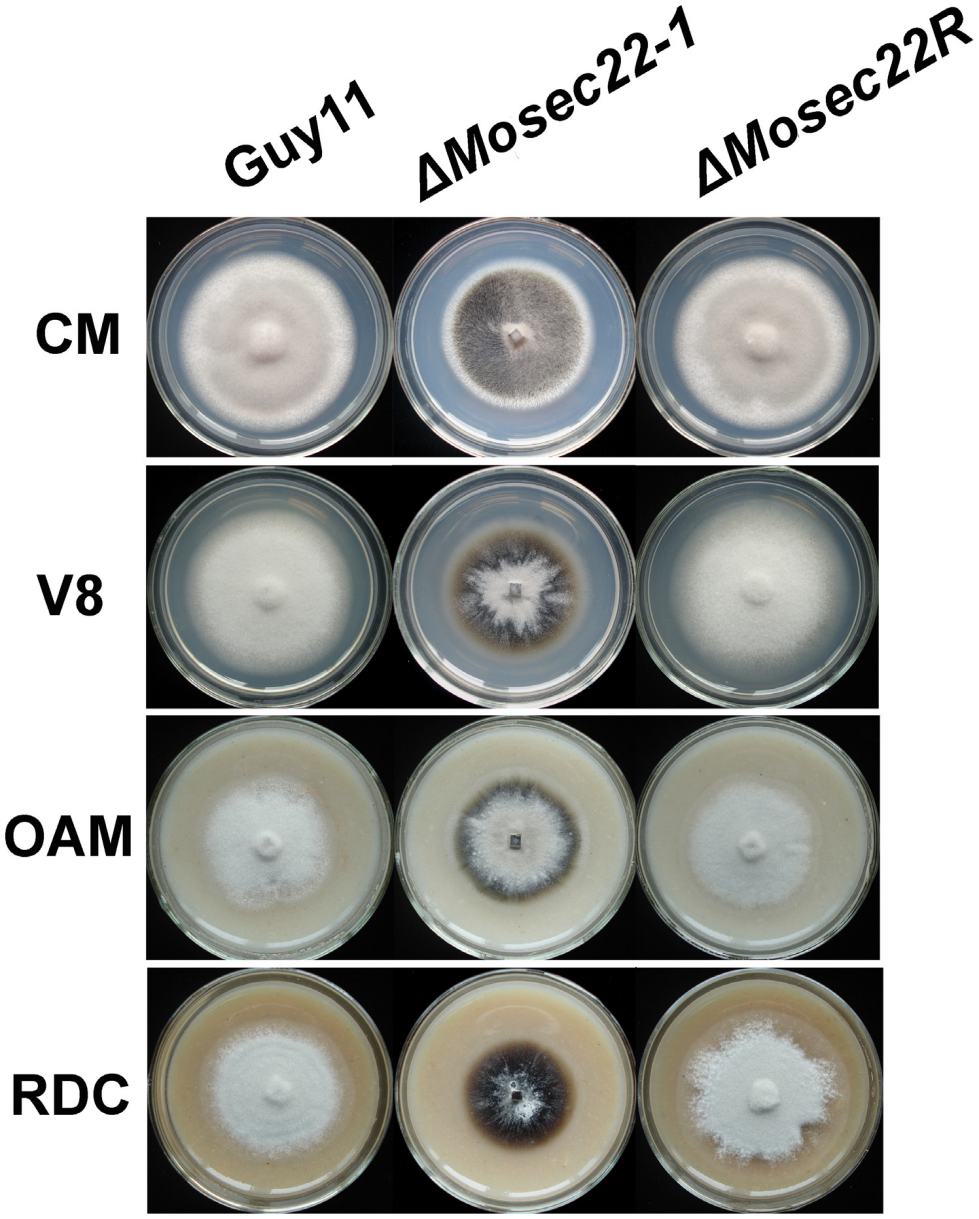
*MoSEC22* disruption resulted in reduced growth. The Δ*Mosec22* (#1) mutant displayed growth reduction on CM, V8, OMA, and RDC medium. And the Δ*Mosec22* (#2) mutant shared the same phenotype with #1. Data comprise three independent experiments with triple replications each time that yielded similar results.

The Δ*Mosec22* mutant showed reduced vegetative growth in comparison to the wild-type strain Guy11 on CM, V8, OMA and RDC medium. The Δ*Mosec22* mutants lacked aerial hyphae and underwent progressive autolysis on conidiation RDC medium leaving eventually a water-soaked film. The mycelia resumed growth after transferring of agar blocks from completely lysed plates to fresh liquid CM, indicating that parts of the fungal hyphae survived autolysis (Figure 3).

**Figure 4:**
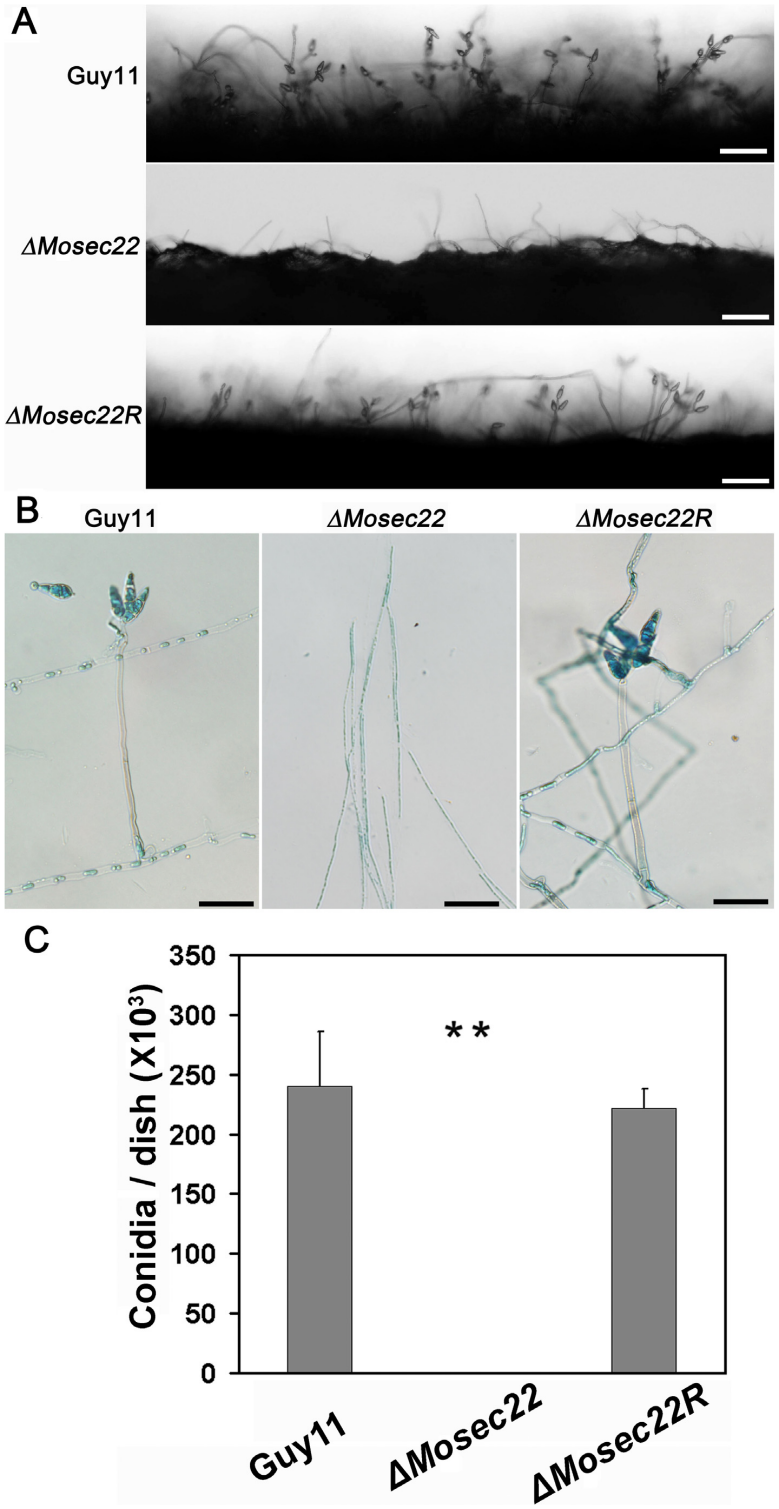
*MoSEC22* disruption affects conidiophore formation. (A) Development conidia on conidiophores. Light microscopic observation was performed on strains grown on RDC medium for 9 days. Bars  = 100 µm. (B) Aerial structures stained with lactophenol aniline blue. Conidia and aerial hyphae stained blue, and conidiophores stained gray. Bars  = 30 µm. (C) The Δ*Mosec22* (#1) mutant produced few conidia in comparison to Guy11 and Δ*Mosec22R*. Per dish sporulation of wild type Guy11, Δ*Mosec22* (#1), and Δ*Mosec22R* strains are indicated. Values indicate standard deviations from the means. Asterisks indicate a significant difference between the sporulation of the mutants and wild type strain (or reconstituted strain) at p = 0.01, according to the Duncan's range test.

Quantitative measurements confirmed that production of conidia was completely abolished in the Δ*Mosec22* mutant on CM, V8, OMA or RDC medium. In addition, no conidia were observed in the Δ*Mosec22* mutant after prolonged incubation under conidial induction conditions (Figure 4). We further analysis the conidiophore differentiation and conidial formation in the mutant. As shown in Figure 4A, no conidiophore was observed in the Δ*Mosec22* mutant at 24 h post conidial induction after 8-day old incubation. Moreover even at 48 h post induction few conidiopores were differentiated. However, the wild-type and Δ*Mosec22R* developed pearshaped conidia on a normal conidiophore. In order to determine if MoSec22 is involved in conidiophore development, staining with lactophenol aniline blue was used to distinguish conidiophores from other aerial hyphae [32]. Microscopic examination revealed that no conidiophores developed in the Δ*Mosec22* mutant (Figure 4B). These data suggested that MoSec22 is required for conidiophore formation.

**Figure 5:**
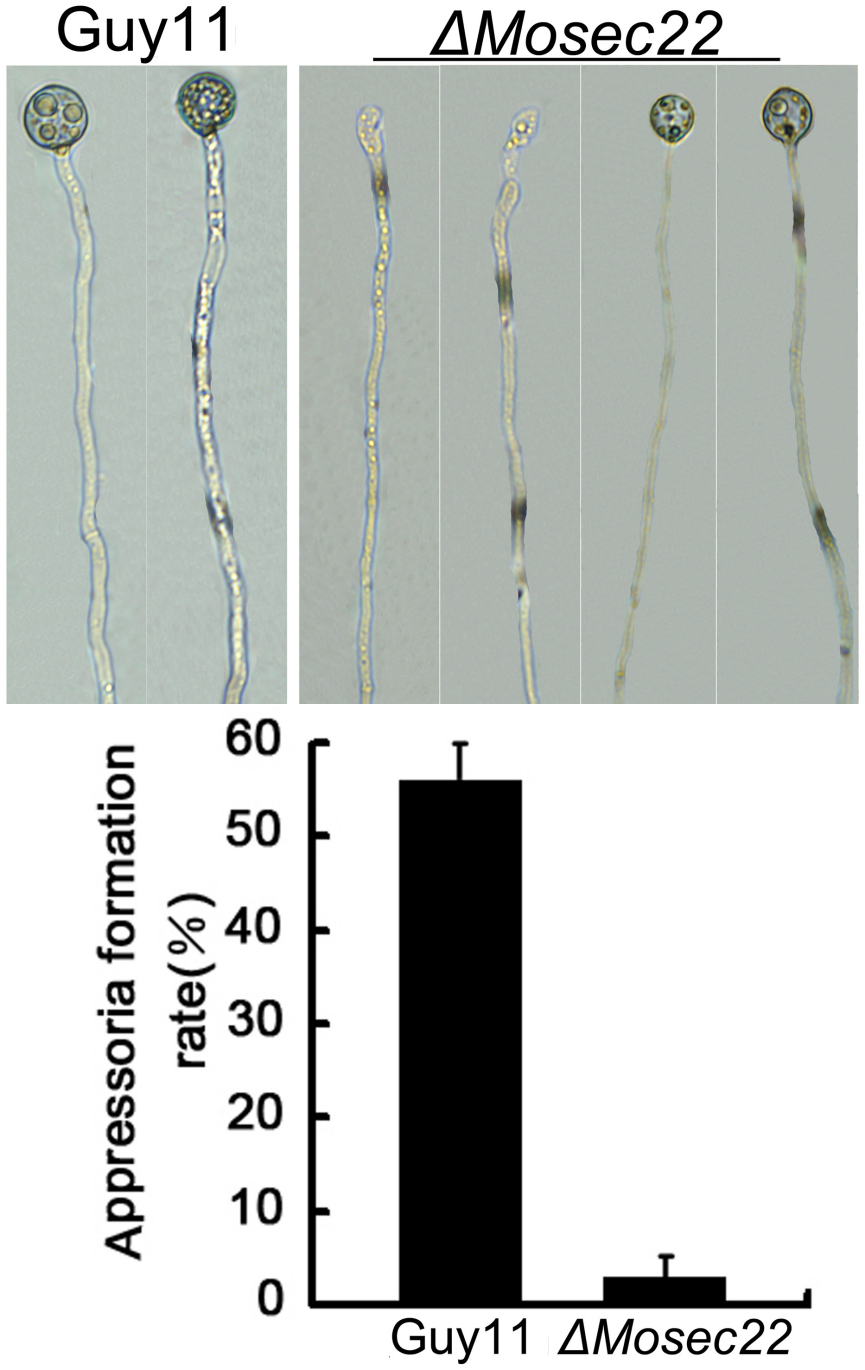
Appressorium formation was blocked by *MoSEC22* disruption. The fragmented mycelia suspension were incubated on the surface of hydrophobic Gelbond film and the rates of appressorium formation were calculated at 12 and 24 hours post incubation. Data consisted of three independent experiments with triple replications that yielded similar results.

To evaluate the role of MoSec22 in appressorium formation, actively growing hyphal suspension was placed on an inductive hydrophobic surface of Gelbond film or onion epidermal cells. Microscopic examination revealed that the Δ*Mosec22* mutant sparsely formed appressoria at the hyphal tips, in contrast to the wild-type strain that formed appressoria on 55% of hyphal tips (Figure 5). In addition, the appressoria of Δ*Mosec22* were smaller in size than those of the wild type strain (Figure 5). These results suggest that MoSec22 plays a critical role in appressorium formation.

### Δ*Mosec22* mutants showed loss of pathogenicity

**Figure 6:**
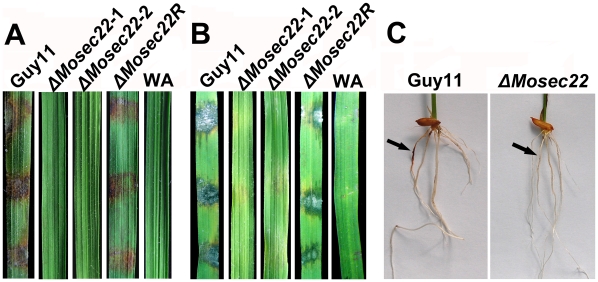
Pathogenicity assay on rice leaves and roots. (A) and (B) Pathogenicity assays on rice (*Oryza sativa* cv. CO39). Rice leaves unwounded (A) and wounded (B) were inoculated with wild type Guy11, Δ*Mosec22*, and reconstituted Δ*Mosec22* (Δ*Mosec22*R) strains with water agar as control (WA). Representing leaves were photographed 5 days post inoculation. The experiments were repeated at least three times with triple replications that yielded similar results. (C) Blast symptoms on rice roots. Arrows show necrotic lesions.

*M. oryzae* enters rice leaves and stems primarily through the appressorium, a specialised structure that develops from a germinated conidium. However, hyphae can also invade rice roots [40] and wounded leaf tissues [41]. As the Δ*Mosec22* deletion mutants hardly produced any conidia in the present study, we inoculated mycelial plugs of the deletion mutant onto wounded and non-wounded rice leaves. No disease symptoms developed on either wounded or non-wounded leaves infected with Δ*Mosec22* mutant plugs 5 days post inoculation, in contrast to leaves infected with wild-type Guy11 and *MoSEC22* complement mutant Δ*Mosec22R* strains that developed typical rice blast lesions (Figure 6A and 6B). We further examined the pathogenicity of the mutant in a root infection assay and the result recapitulated that the Δ*Mosec22* disruption mutant was avirulent (Figure 6C). These observations indicated that MoSec22 is essential for the pathogenicity of *M. oryzae*.

### Cell wall integrity is altered in the ΔMosec22 mutant

**Figure 7:**
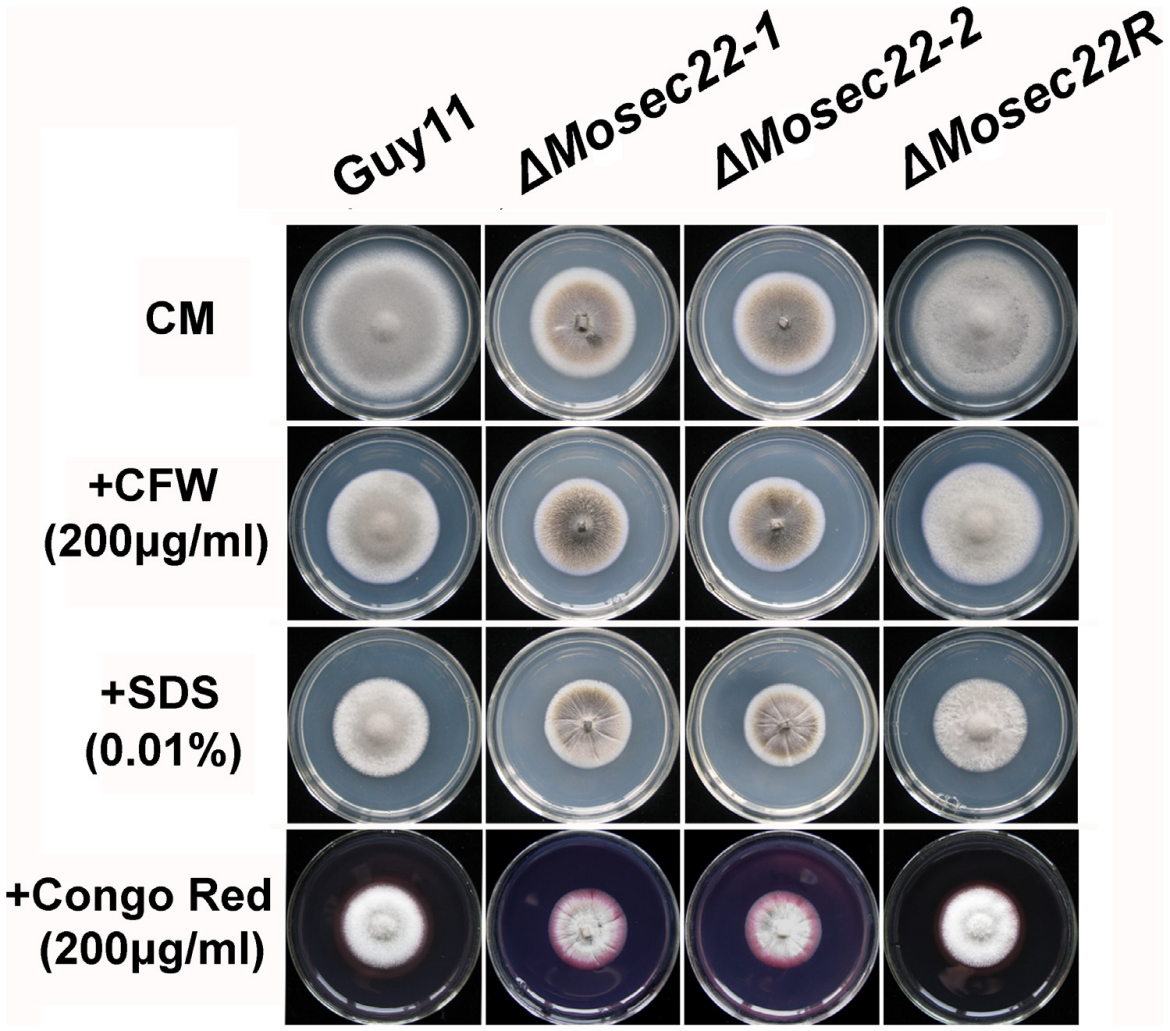
MoSec22 is involved in the tolerance of cell wall or membrane to stress inducers. The Guy11, Δ*Mosec22* mutants (#1 and #2), and the reconstitute strain (Δ*Mosec22R*) were incubated on CM medium supplemented with, respectively, 200 µg/ml CFW, 0.01% SDS, and 200 µg/ml CR at 28°C for 7 days before being photographed. Data comprise three independent experiments with triple replications each time that yielded similar results.

To investigate the cause of defects resulting from loss of MoSec22, the structural integrity of the cell wall and membrane of the Δ*Mosec22* mutant was examined (Table S2). Mycelial growth was measured on CM medium containing various concentrations of the cell wall stressors CFW, SDS, and Congo Red (CR). As CFW binds to chitin, interfering with its polymerisation [42], [43], CFW sensitivity test is often used to identify mutants defective in cell wall assembly or in signal transduction regulating cell wall integrity [44], [45], [46]. In growth assays, Δ*Mosec22* mutant showed less resistance to CFW than the wild type as well as the reconstituted strains (Figure 7), suggesting that MoSec22 was involved in maintaining the integrity of the cell wall. SDS is a detergent that reduces membrane stability and any cell wall defects will lead to increased accessibility of SDS to the plasma membrane resulting sensitivity [47], [48], [49]. The Δ*Mosec22* mutants were also less resistant to SDS compared with wild-type Guy11 and Δ*Mosec22R* strains (p<0.01). Furthermore, we tested the third cell wall perturbing agent CR [50] by observing the growth of strains on CR containing medium. The result, in which both Δ*Mosec22* mutants exhibited smaller diameter in colony sizes and less dense aerial hyphae, was similar to those of above, all indicating more inhibition on the growth of the Δ*Mosec22* mutant than the wild-type strain by those agents (Figure 7).

**Figure 8:**
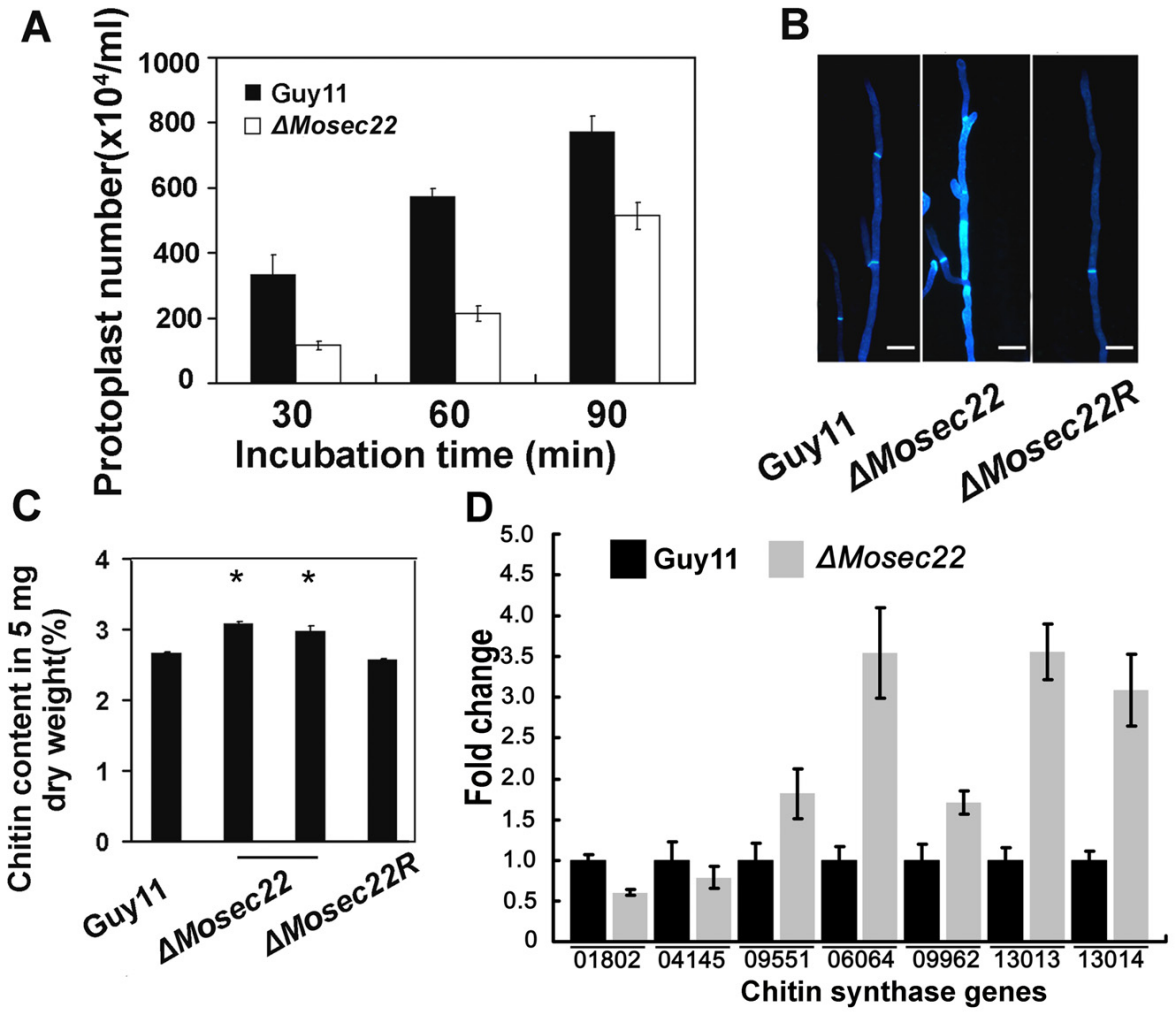
Deletion of *MoSEC22* alters the fungal cell wall. (A) Protoplasts released under the treatment of cell-wall-degrading enzymes. The released protoplast was quantified at 30 min intervals. Data comprise three independent experiments, with triple replications each time yielding similar results. (B) Disruption of *MoSEC22* (#1) altered the distribution of chitin on the cell wall. The experiment was repeated several times with triple replications that yielded similar results. (C) GlcNa determination by the fluorimetric Morgan–Elson method shows increased chitin contents in the Δ*Mosec22* mutant. Asterisks indicate a significant difference between the sporulation in the mutant and wild-type strains (or the reconstituted strain) at *p* = 0.01, according to Duncan's range test. Data comprise three independent experiments with triple replications each time that yielded similar results. (D) Transcription analysis of seven *M. oryzae* chitin synthases using qRT-PCR.

Finally, we examined the effects of lytic enzymes (10 mg/ml lysing enzymes) to the Δ*Mosec22* mutant. Interestingly, less protoplasts were found in the Δ*Mosec22* mutant than the controls (Figure 8A), suggesting that either the altered cell wall structure rending it less accessible to or resistant to lytic enzymes, or excess rupture and poor recovery of protoplasts due to breached membrane and cell walls. Judging from above tests using cell wall perturbing agents, the latter scenario is very likely.

### ΔMosec22 mutant increased cell wall chitin deposition

In *S. cerevisiae*, Sec22 is involved in the regulation of chitin synthesis [51]. To determine whether MoSec22 has a similar role, we examined the cell wall properties of the Δ*Mosec22* mutant using chitin stain CFW. In the wild-type strain Guy11, newly synthesised chitin indicated by CFW fluorescence was mostly distributed at the septa and tips where it was actively synthesised, while the bright fluorescence was not restricted to growing apices but also found on lateral walls along hyphal axes in the mutants (Figure 8B). This abnormal distribution of cell wall components was restored by re-introduction of the wild-type *MoSEC22* gene. This abnormal distribution of cell wall components was restored by re-introduction of the wild-type *MoSEC22* gene. It is well documented that the synthesis of Chitin, which is a constituent of fungal cell wall, depends on the activity of the chitin synthase enzymes, and they are wildly existed in many fungal species including *M. oryzae*. These enzymes could be divided into seven classes (classes I-VII) [52], [53]. Class I, II and V chitin synthase have been proved to be involved in chitin synthesis in the mycelium of *Fusarium oxysporum* and *Botrytis cinerea* [54], [55]. However, in *M. oryzae*, the function of these homologious genes, except for class VII chitin synthase enzyme encoding genes [52], is still unclear to date. Combined with results above, we further quantified the accumulation of chitin and examined the expression of several genes known to be involved in chitin synthesis. The chitin contents were increased by 20% in Δ*Mosec22* mutant relative to the wild-type strain (Figure 8C), and consistently, the transcription of five out of seven genes were increased (Figure 8D).

### Loss of MoSEC22 reduced the accumulation of reactive oxygen species *in vivo*

**Figure 9:**
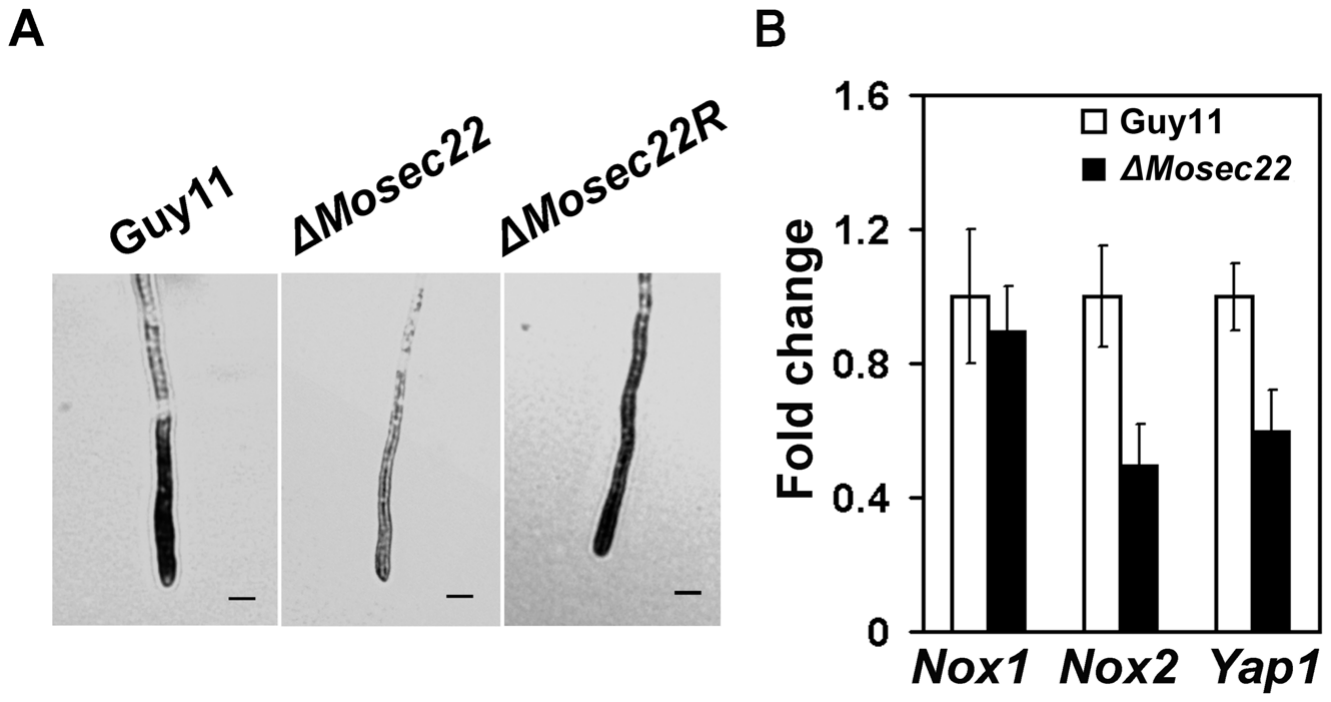
ROS levels and the expression of the relevant genes. (A) Dark NBT dye in wild type Guy11 strain suggests a high ROS level, whereas Δ*Mosec22* mutants displayed less color, indicating a reduced ROS accumulation. The reconstituted strain exhibited a darker color similar to Guy11. All strains were grown for 2 days at room temperature on CM-overlaid microscope slides before staining. The experiments were repeated at least three times with similar results. (B) Fold change of the genes related to ROS by quantitative Real-Time PCR.

Previous studies indicated that reactive oxygen species (ROS) were essential for fungal pathogenicity [56]. ROS were observed at distinct times during conidial germination, appressorium development and hyphal tip growth in *M. oryzae* [57]. Since the disruption of Δ*Mosec22* resulting in defects in hyphal growth, sporulation, appressorium formation, and pathogenicity, we measured the intracellular ROS accumulation at the hyphal tips using nitroblue tetrazolium (NBT), which forms a dark-blue water-insoluble formazan precipitate upon reduction by superoxide radicals. The Δ*Mosec22* mutant generated significantly less superoxide than Guy 11 during mycelial growth, as quantified by a reduction in the mean pixel intensity measurement due to accumulation of formazan precipitate (Figure 9A), suggesting that compromised superoxide accumulation was partially responsible for the low ROS level.

ROS are generated primarily by NADPH oxidases localised at the plasma membrane [58]. In *M. oryzae*, Nox1 and Nox2 are important sources of ROS production [57]. Therefore, we performed real-time quantitative PCR analysis to examine the expression of *NOX1* and *NOX2* and found that the transcript levels were reduced by 20% and 50%, respectively, relative to the wild-type strain (Figure 9B). Since the expression of genes involved in the detoxification of ROS is regulated by the transcription factor Yap1 [59], we also examined the transcription of a *M. oryzae* Yap1 homologue (MGG_12814.6) and found that its transcript was reduced to 56% in the Δ*Mosec22* mutant (Figure 9B). Together, these findings indicate that disruption of MoSec22 has a negative impact directly and indirectly on the membrane function.

### Disruption of *MoSEC22* affected extracellular laccase and peroxidase activities

**Figure 10:**
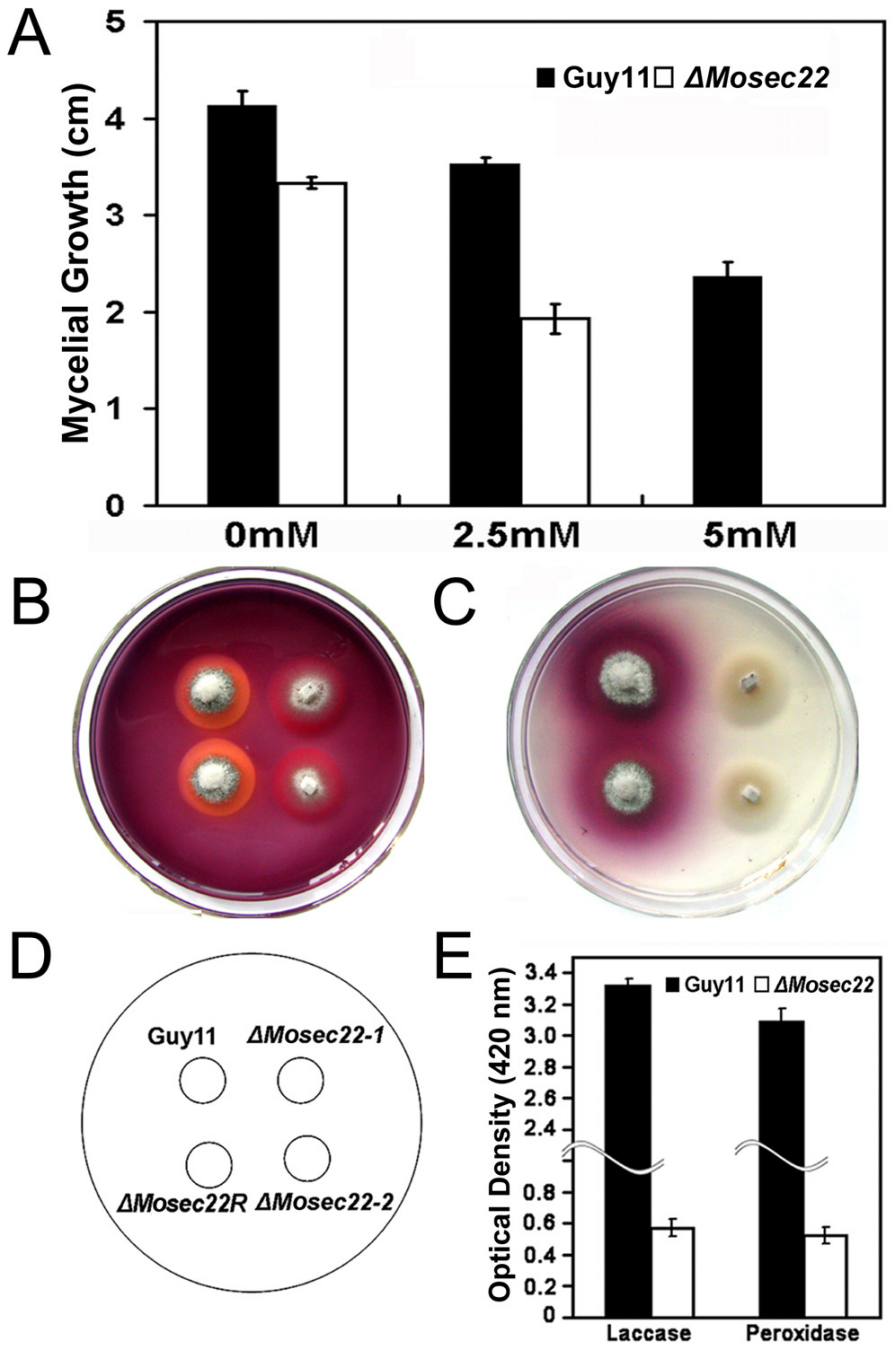
Compromised activities of extracellular peroxidases and laccases in Δ*Mosec22* mutants. (A) Δ*Mosec22* mutant is hypersensitive to H_2_O_2_. The Guy11, Δ*Mosec22* mutant (#1) were incubated on CM medium supplemented with 2.5 and 5 mM H_2_O_2_ for 6 days before being photographed. (B) Strains of Guy11 and Δ*Mosec22* mutants were inoculated on CM agar medium containing 200 µg/ml CR. Discoloration was observed after 5 days. (C) The laccase activity was monitored in complete media supplemented with 0.2 mM ABTS 3 days after inoculation. Three independent experiments with triplicate replicates were performed. (D) The positions of Guy11, Δ*Mosec22-*#1, Δ*Mosec22-*#2, and Δ*Mosec22R* showed on CM plates in B and C are indicated. E) The laccase and peroxidase activities were measured by the ABTS oxidization test without or with H_2_O_2_. In all experiments, three independent experiments were carried out with triplicate replicates each time. Error bars represent the standard deviations where applicable.

**Figure 11:**
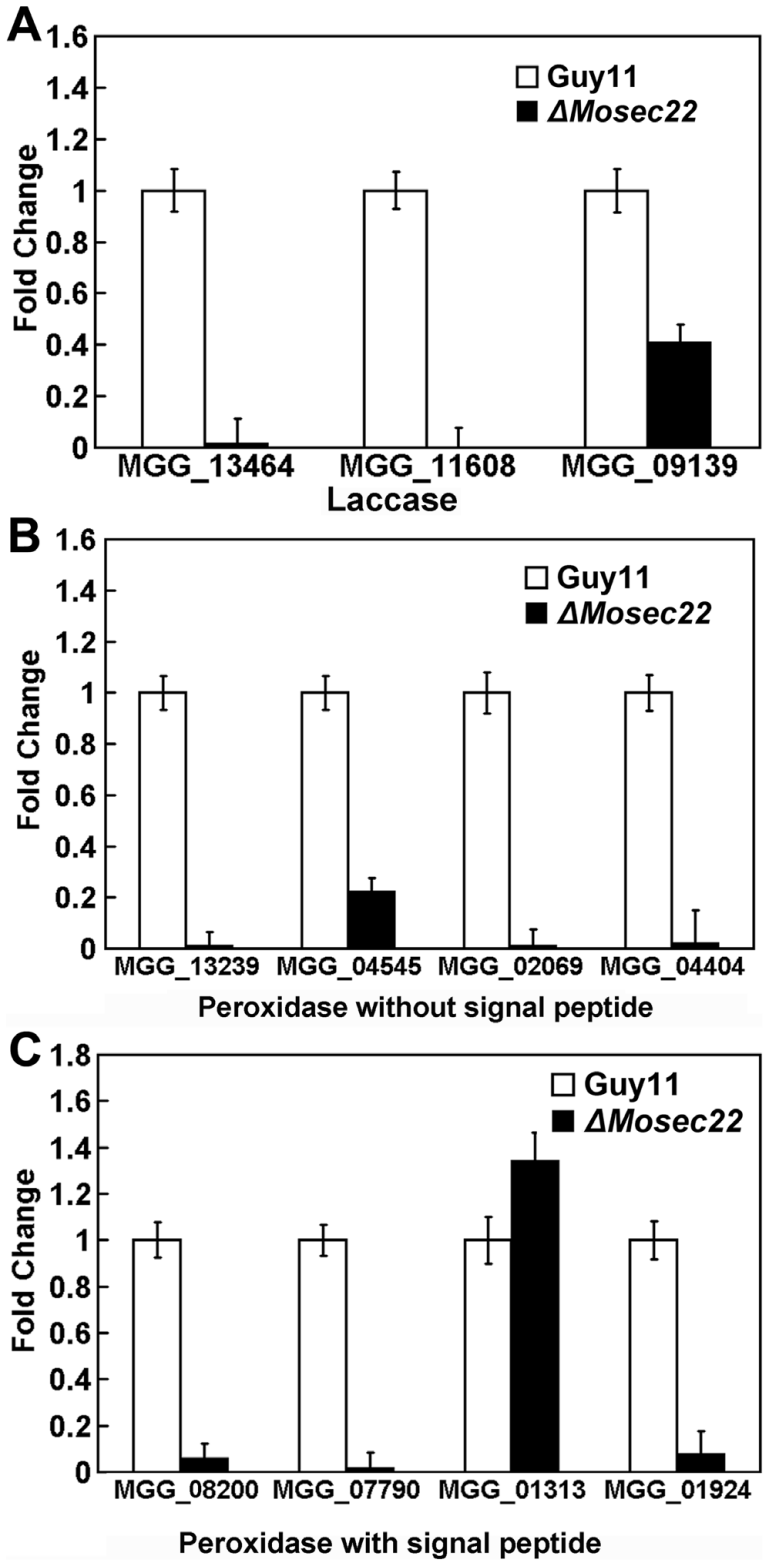
The expression profile of laccase and peroxidases. (A) Expression profiles of putative laccase-encoding genes in the Δ*Mosec22* mutant. The transcript levels of the putative laccase-encoding genes in both the Δ*Mosec22* mutant and the wild type strains are obtained from three independent experiments. Error bars represent the standard deviations. (B) and (C) The expression of five genes encoding predicted peroxidases without (B) or with (C) a signal peptide domain. The transcript levels are indicated as the means from three independent experiments. Error bars represent the standard deviations.

In this study, we found that the mycelial growth of the Δ*Mosec22* mutant was markedly reduced in the presence of both 2.5 mM and 5 mM hydrogen peroxide (H_2_O_2_) in comparison to controls (Figure 10A). A recent study in *M. oryzae*, which ascribed the oxidative sensitivity to the decreased activity of the extracellular peroxidases [37], suggested that MoSec22 may participate in the degradation of extracellular ROS such as H_2_O_2_. CR is also utilized as an indicator for the presence of secreted peroxidase. Thus, Δ*Mosec22* mutants were inoculated onto CR-containing media. No significant differences in mycelial growth were observed initially, however, a bright degradation halo was later developed surrounding the wild-type strain but not the Δ*Mosec22* mutant (Figure 10B), indicating reduced extracellular peroxidase activities in the latter strain. The filtrates of the Δ*Mosec22* mutant as well as the Guy11 strain were collected and extracellular peroxidase activities measured by use of a colorimetric assay. The results indicated an almost complete loss of the peroxidase activity in the Δ*Mosec22* mutant (Figure 10E). The activities of additional extracellular enzyme, laccase, were also measured for cells grown on solid medium and in the culture filtrate, which showed that Δ*Mosec22* mutant has a decreased laccase activity, as indicated by the difference in the degree of the oxidised dark purple reaction (Figure 10C) and the laccase activity (Figure 10E). Moreover, real-time quantitative PCR analysis indicated that the transcript levels of several peroxidases (MGG_08200, MGG_07790, MGG_01924, MGG_13239, MGG_04545, MGG_02069, and MGG_04404) and laccases (MGG_13464, MGG_11608, and MGG_09139) were all markedly down in the Δ*Mosec22* deletion mutant (Figure 11A, 11B and 11C). Collectively, these findings indicate that the sensitivity of the Δ*Mosec22* mutant to stress inducing agents such as H_2_O_2_ is due to its reduced production/secretion of extracellular peroxidases and laccases.

## Discussion

In the present study, we characterised a SNARE homolog protein, MoSec22, in *M. oryzae* and primarily focused on its external phenotypes associated with pathogenesis. Gene-targeted replacement revealed that the loss of MoSec22 led to a plethora of developmental defects, and further analysis showed that MoSec22 was involved in the maintenance of cell wall integrity and ROS generation. Whereas the detailed roles of MoSec22 in membrane fusion and trafficking remains to be investigated, our current data suggest that the SNARE proteins are likely carrying essential roles in this fungus.

Conidiogenesis and appressorium development play critical roles in the rice blast disease cycle. These processes are governed by a precise developmental programme in response to stimuli from the host and the environment. This fungus has evolved regulatory networks to ensure the correct timing and spatial pattern of these development events [37]. Recently, we found that the SNARE protein MoVAM7 participates in conidiogenesis and appressorium development (Dou & Zhang, unpublished data). Here, the Δ*Mosec22* mutant hardly produced conidia or appressoria at the tips of the hyphae, all suggesting that conidiogenesis and appressorium formation are regulated at many levels and proteins including those functioning in membrane trafficking. In many fungi, it is well documented that the aerial hyphal formation plays a significant role during conidiophores differentiation and asexual spores production [60], [61], [62], [63]. However, by the assay of the conidiation, we found the autolysis of the Δ*Mosec22* mutants on RDC medium, indicating that the lost ability of conidiation of the mutant may be due to the lost ability of formation of conidiophores, which were ascribed to autolysis of the aerial hyphal of the Δ*Mosec22* mutants on conidiation medium.

Fungal cell walls are composed of a tight, semi permeable fibrillar network of polymers, including chitin, glucan composed of polysaccharides and mannoproteins [64]. In the present study, the Δ*Mosec22* deletion mutants showed increased sensitivity to cell wall stressors. Further analysis indicated an increase in the chitin biosynthesis activity and abnormal accumulation of chitin. This may indicate that a negative feedback regulatory circuitry exists for chitin synthesis. Chitin is an integral part of the fungal cell wall and the synthesis of chitin depends on the activity of chitin synthase enzymes, such as Chs3p of *S. cerevisiae*, which accounts for about 90% of chitin synthesis activities. Chs3p transits through the ER/Golgi secretory pathway to the plasma membrane early in the process of daughter cell formation. Once the daughter cell has reached its full size, Chs3p is retrieved by endocytosis into intracellular “chitosomes” [36]. In *U. maydis*, a genome-wide localisation analysis of all chitin synthases using GFP fusion proteins indicated that some of these chitin synthases were co-localized with the endosomes, suggesting that chitin synthases are good candidates for recycling processes at the growing hyphal tips [13]. It has also been speculated that the polarized growth of filamentous fungi requires the endocytic uptake and recycling of cell wall components such as chitin synthases. Consistent with this the FM4-64 staining revealed that endocytosis was blocked by disruption of *MoSEC22* (see Figure S1). Failure to recycle due to *MoSEC22* disruption may reduce the levels of chitin synthases available for further rounds of vesicle fusion with the target membrane, and this lack of vesicular SNARE may also change the polarised transport of cell wall components or cell wall synthesis-related enzymes resulting in multiple defects in the Δ*Mosec22* mutant.

ROS is important in the processes of plant infection and rice blast disease [65]. A Rac GTPase was implicated in the activation of the NADPH oxidase and also in inhibition of the expression of the ROS scavenger metallothionein during defence signalling [66]. In *M. oryzae*, a *Morac1* deletion mutant is defective in conidia production [67]. Consistent with these previous reports, the lower ROS levels resulted from *MoSEC22* disruption may partly contribute to the defects of Δ*Mosec22* mutants. As NADPH oxidase (Nox) proteins are responsible for ROS production [68] and Nox1 and Nox2 are important in pathogenesis [57], our findings of the downregulation in the expression of both Nox1 and Nox2 in Δ*Mosec22* may provide a clue for the decreased ROS levels and attenuated virulence.

In *U. maydis*, the transcription factor Yap1 controls the expression of peroxidase genes (um01947 and um10672) and is responsible for scavenging host-derived ROS in its interaction with the plant [69]. We found that the expression of MoYap1 was decreased in the Δ*Mosec22* mutant and that MoYap1 is involved in tolerance to H_2_O_2_ and virulence (Guo and Zhang, unpublished data), suggesting a functional link between MoSec22 and MoYap1.

MoSec22 is important in the accumulation of extracellular peroxidases that are known to play the crucial roles in scavenging ROS, which accumulates rapidly in the major and earliest responses of plant pathogen-associated molecular pattern (PAMP)-triggered immunity (PTI) [70]. In *M. oryzae*, the *des1* deletion mutant displays decreased expression of peroxidase genes, and this severely affects the virulence on susceptible rice cultivar *Nakdongbyeo* [37]. Recently, we reported that the transcription factor MoATF1 of *M. oryzae* is responsible for the scavenging of host-derived ROS, and that this function may be essential for the inhibition of plant defense response and the spread of infection hyphae in plant tissues [30]. Defects in production of these extracellular peroxidases resulting in loss of pathogenicity may also cause loss of inhibition of the plant PTI response.

In summary, we found that MoSec22 is a multifunctional protein required for conidiogenesis and pathogenicity. MoSec22 participates in the maintenance of cell wall integrity and regulation of ROS levels. Each and any of these processes are crucial for the growth, development, and pathogenicity of the fungus. How MoSec22 exerts these functions, directly or indirectly through being a component of the endomembrane system necessitates further studies.

## Supporting Information

Table S1 The primers used in this study.
(0.06 MB DOC)

Table S2 The *ΔMosec22* mutant displays increased sensitivity to cell wall perturbing agents.
(0.03 MB DOC)

Figure S1 FM4-64 staining reveals that the *ΔMosec22* mutant is defective in endocytosis.
(3.11 MB PPT)

## References

[1] Chen YA, Scheller RH (2001). SNARE-mediated membrane fusion.. Nat Rev Mol Cell Biol.

[2] Fasshauer D, Eliason WK, Brunger AT, Jahn R (1998). Identification of a minimal core of the synaptic SNARE complex sufficient for reversible assembly and disassembly.. Biochemistry.

[3] Hong W (2005). SNAREs and traffic.. Biochim Biophys Acta.

[4] Jahn R, Lang T, Sudhof TC (2003). Membrane fusion.. Cell.

[5] Jahn R, Scheller RH (2006). SNAREs—engines for membrane fusion.. Nat Rev Mol Cell Biol.

[6] Pelham HR (1999). SNAREs and the secretory pathway-lessons from yeast.. Exp Cell Res.

[7] Rothman JE (1994). Mechanisms of intracellular protein transport.. Nature.

[8] Burri L, Lithgow T (2004). A complete set of SNAREs in yeast.. Traffic.

[9] Burri L, Varlamov O, Doege CA, Hofmann K, Beilharz T (2003). A SNARE required for retrograde transport to the endoplasmic reticulum.. Proc Natl Acad Sci U S A.

[10] Sanderfoot AA, Assaad FF, Raikhel NV (2000). The *Arabidopsis* genome. An abundance of soluble N-ethylmaleimide-sensitive factor adaptor protein receptors.. Plant Physiol.

[11] Gupta GD, Brent Heath I (2002). Predicting the distribution, conservation, and functions of SNAREs and related proteins in fungi.. Fungal Genet Biol.

[12] Kuratsu M, Taura A, Shoji JY, Kikuchi S, Arioka M (2007). Systematic analysis of SNARE localization in the filamentous fungus *Aspergillus oryzae*.. Fungal Genet Biol.

[13] Fuchs U, Steinberg G (2005). Endocytosis in the plant-pathogenic fungus *Ustilago maydis*.. Protoplasma.

[14] Wedlich-Soldner R, Bolker M, Kahmann R, Steinberg G (2000). A putative endosomal t-SNARE links exo- and endocytosis in the phytopathogenic fungus *Ustilago maydis*.. EMBO J.

[15] Talbot NJ (2003). On the trail of a cereal killer: Exploring the biology of *Magnaporthe grisea*.. Annu Rev Microbiol.

[16] Ebbole DJ (2007). *Magnaporthe* as a model for understanding host-pathogen interactions.. Annu Rev Phytopathol.

[17] Thompson JD, Higgins DG, Gibson TJ (1994). CLUSTAL W: improving the sensitivity of progressive multiple sequence alignment through sequence weighting, position-specific gap penalties and weight matrix choice.. Nucleic Acids Res.

[18] Dean RA, Talbot NJ, Ebbole DJ, Farman ML, Mitchell TK (2005). The genome sequence of the rice blast fungus *Magnaporthe grisea*.. Nature.

[19] Skamnioti P, Gurr SJ (2007). *Magnaporthe grisea* cutinase2 mediates appressorium differentiation and host penetration and is required for full virulence.. Plant Cell.

[20] Gilbert MJ, Thornton CR, Wakley GE, Talbot NJ (2006). A P-type ATPase required for rice blast disease and induction of host resistance.. Nature.

[21] Yi M, Chi MH, Khang CH, Park SY, Kang S (2009). The ER chaperone LHS1 is involved in asexual development and rice infection by the blast fungus *Magnaporthe oryzae*.. Plant Cell.

[22] Cao X, Barlowe C (2000). Asymmetric requirements for a Rab GTPase and SNARE proteins in fusion of COPII vesicles with acceptor membranes.. J Cell Biol.

[23] Spang A, Schekman R (1998). Reconstitution of retrograde transport from the Golgi to the ER in vitro.. J Cell Biol.

[24] Ni L, Snyder M (2001). A genomic study of the bipolar bud site selection pattern in *Saccharomyces cerevisiae*.. Mol Biol Cell.

[25] Steinmetz LM, Scharfe C, Deutschbauer AM, Mokranjac D, Herman ZS (2002). Systematic screen for human disease genes in yeast.. Nat Genet.

[26] Zhang H, Zhao Q, Liu K, Zhang Z, Wang Y (2009). MgCRZ1, a transcription factor of *Magnaporthe grisea*, controls growth, development and is involved in full virulence.. FEMS Microbiol Lett.

[27] Tamura K, Dudley J, Nei M, Kumar S (2007). MEGA4: Molecular Evolutionary Genetics Analysis (MEGA) software version 4.0.. Mol Biol Evol.

[28] Gietz RD, Schiestl RH, Willems AR, Woods RA (1995). Studies on the transformation of intact yeast cells by the LiAc/SS-DNA/PEG procedure.. Yeast.

[29] Talbot NJ, Ebbole DJ, Hamer JE (1993). Identification and characterization of MPG1, a gene involved in pathogenicity from the rice blast fungus *Magnaporthe grisea*.. Plant Cell.

[30] Guo M, Guo W, Chen Y, Dong S, Zhang X (2010). The basic leucine zipper transcription factor Moatf1 mediates oxidative stress responses and is necessary for full virulence of the rice blast fungus *Magnaporthe oryzae*.. Mol Plant Microbe Interact.

[31] Livak KJ, Schmittgen TD (2001). Analysis of relative gene expression data using real-time quantitative PCR and the 2(-Delta Delta C(T)) Method.. Methods.

[32] Zhou Z, Li G, Lin C, He C (2009). *Conidiophore stalk-less1* encodes a putative zinc-finger protein involved in the early stage of conidiation and mycelial infection in *Magnaporthe oryzae*.. Mol Plant Microbe Interact.

[33] Harris SD, Morrell JL, Hamer JE (1994). Identification and characterization of *Aspergillus nidulans* mutants defective in cytokinesis.. Genetics.

[34] Zheng W, Chen J, Liu W, Zheng S, Zhou J (2007). A Rho3 homolog is essential for appressorium development and pathogenicity of *Magnaporthe grisea*.. Eukaryot Cell.

[35] Dufresne M, Osbourn AE (2001). Definition of tissue-specific and general requirements for plant infection in a phytopathogenic fungus.. Mol Plant Microbe Interact.

[36] Bulik DA, Olczak M, Lucero HA, Osmond BC, Robbins PW (2003). Chitin synthesis in *Saccharomyces cerevisiae* in response to supplementation of growth medium with glucosamine and cell wall stress.. Eukaryot Cell.

[37] Chi MH, Park SY, Kim S, Lee YH (2009). A novel pathogenicity gene is required in the rice blast fungus to suppress the basal defenses of the host.. PLoS Pathog.

[38] Liu Y, Flanagan JJ, Barlowe C (2004). Sec22p export from the endoplasmic reticulum is independent of SNARE pairing.. J Biol Chem.

[39] Slack JK, Lowry CV, Carroll AM (1994). HASTEN: a technique to identify the primary structure of terminal DNA hairpins.. Nucleic Acids Res.

[40] Sesma A, Osbourn AE (2004). The rice leaf blast pathogen undergoes developmental processes typical of root-infecting fungi.. Nature.

[41] Silu D, Tharreau D, Talbot NJ, Clergeot PH, Notteghem JL (1998). Identification and characterization ofapf1-in a non-pathogenic mutant of the rice blast fungus *Magnaporthe grisea* which is unable to differentiate appressoria.. Physiological and Molecular Plant Pathology.

[42] Roncero C, Duran A (1985). Effect of Calcofluor white and Congo red on fungal cell wall morphogenesis: in vivo activation of chitin polymerization.. J Bacteriol.

[43] Elorza MV, Rico H, Sentandreu R (1983). Calcofluor white alters the assembly of chitin fibrils in *Saccharomyces cerevisiae* and *Candida albicans* cells.. J Gen Microbiol.

[44] Lussier M, White AM, Sheraton J, di Paolo T, Treadwell J (1997). Large scale identification of genes involved in cell surface biosynthesis and architecture in *Saccharomyces cerevisiae*.. Genetics.

[45] Ram AF, Brekelmans SS, Oehlen LJ, Klis FM (1995). Identification of two cell cycle regulated genes affecting the beta 1,3-glucan content of cell walls in *Saccharomyces cerevisiae*.. FEBS Lett.

[46] Ram AF, Wolters A, Ten Hoopen R, Klis FM (1994). A new approach for isolating cell wall mutants in *Saccharomyces cerevisiae* by screening for hypersensitivity to calcofluor white.. Yeast.

[47] Bickle M, Delley PA, Schmidt A, Hall MN (1998). Cell wall integrity modulates RHO1 activity via the exchange factor ROM2.. EMBO J.

[48] Igual JC, Johnson AL, Johnston LH (1996). Coordinated regulation of gene expression by the cell cycle transcription factor Swi4 and the protein kinase C MAP kinase pathway for yeast cell integrity.. EMBO J.

[49] Shimizu J, Yoda K, Yamasaki M (1994). The hypo-osmolarity-sensitive phenotype of the *Saccharomyces cerevisiae* hpo2 mutant is due to a mutation in PKC1, which regulates expression of beta-glucanase.. Mol Gen Genet.

[50] Wood PJ, Fulcher RG (1983). Dye interactions. A basis for specific detection and histochemistry of polysaccharides.. J Histochem Cytochem.

[51] Lesage G, Shapiro J, Specht CA, Sdicu AM, Menard P (2005). An interactional network of genes involved in chitin synthesis in *Saccharomyces cerevisiae*.. BMC Genet.

[52] Odenbach D, Thines E, Anke H, Foster AJ (2009). The *Magnaporthe grisea* class VII chitin synthase is required for normal appressorial development and function.. Mol Plant Pathol.

[53] Choquer M, Boccara M, Gonçalves IR, Soulié MC, Vidal-Cros A (2004). Survey of the *Botrytis cinerea* chitin synthase multigenic family through the analysis of six euascomycetes genomes.. European Journal of Biochemistry.

[54] Madrid MP, Di Pietro A, Roncero MI (2003). Class V chitin synthase determines pathogenesis in the vascular wilt fungus *Fusarium oxysporum* and mediates resistance to plant defence compounds.. Mol Microbiol.

[55] Martin-Udiroz M, Madrid MP, Roncero MI (2004). Role of chitin synthase genes in *Fusarium oxysporum*.. Microbiology.

[56] Segmuller N, Kokkelink L, Giesbert S, Odinius D, van Kan J (2008). NADPH oxidases are involved in differentiation and pathogenicity in *Botrytis cinerea*.. Mol Plant Microbe Interact.

[57] Egan MJ, Wang ZY, Jones MA, Smirnoff N, Talbot NJ (2007). Generation of reactive oxygen species by fungal NADPH oxidases is required for rice blast disease.. Proc Natl Acad Sci U S A.

[58] Doke N, Miura Y, Sanchez LM, Park HJ, Noritake T (1996). The oxidative burst protects plants against pathogen attack: mechanism and role as an emergency signal for plant bio-defence—a review.. Gene.

[59] Moye-Rowley WS (2003). Regulation of the transcriptional response to oxidative stress in fungi: similarities and differences.. Eukaryot Cell.

[60] Adams TH, Wieser JK, Yu JH (1998). Asexual sporulation in *Aspergillus nidulans*.. Microbiol Mol Biol Rev.

[61] Garzia A, Etxebeste O, Herrero-Garcia E, Fischer R, Espeso EA (2009). *Aspergillus nidulans* FlbE is an upstream developmental activator of conidiation functionally associated with the putative transcription factor FlbB.. Mol Microbiol.

[62] Kikuma T, Arioka M, Kitamoto K (2007). Autophagy during conidiation and conidial germination in filamentous fungi.. Autophagy.

[63] Timberlake WE (1991). Temporal and spatial controls of Aspergillus development.. Curr Opin Genet Dev.

[64] Lipke PN, Ovalle R (1998). Cell wall architecture in yeast: new structure and new challenges.. J Bacteriol.

[65] Yukioka H, Inagaki S, Tanaka R, Katoh K, Miki N (1998). Transcriptional activation of the alternative oxidase gene of the fungus *Magnaporthe grisea* by a respiratory-inhibiting fungicide and hydrogen peroxide.. Biochim Biophys Acta.

[66] Valent B, Farrall L, Chumley FG (1991). *Magnaporthe grisea* genes for pathogenicity and virulence identified through a series of backcrosses.. Genetics.

[67] Chen J, Zheng W, Zheng S, Zhang D, Sang W (2008). Rac1 is required for pathogenicity and Chm1-dependent conidiogenesis in rice fungal pathogen *Magnaporthe grisea*.. PLoS Pathog.

[68] Lara-Ortiz T, Riveros-Rosas H, Aguirre J (2003). Reactive oxygen species generated by microbial NADPH oxidase NoxA regulate sexual development in *Aspergillus nidulans*.. Mol Microbiol.

[69] Molina L, Kahmann R (2007). An *Ustilago maydis* gene involved in H_2_O_2_ detoxification is required for virulence.. Plant Cell.

[70] Apostol I, Heinstein PF, Low PS (1989). Rapid Stimulation of an Oxidative Burst during Elicitation of Cultured Plant Cells: Role in Defense and Signal Transduction.. Plant Physiol.

