# Supplementary material for: R-SNARE Homolog MoSec22 Is Required for Conidiogenesis, Cell Wall Integrity, and Pathogenesis of Magnaporthe oryzae
Source: PLoS One. 2010 Oct 6;5(10):e13193. doi: 10.1371/journal.pone.0013193 (PMC2950850; doi:10.1371/journal.pone.0013193)
Supplement: Table S1 — The primers used in this study. (0.06 MB DOC) [file pone.0013193.s001.doc]

Table S1. PCR primer used in this study.

| Primer No. | Oligonucleotide sequence |
| --- | --- |
| FL474 | 5'-TCGACGTCCGAAAGGATCTGT-3' |
| FL475 | 5'-ACTCCTGCTTCGAGATCCACATC-3' |
| FL1111 | 5'-GGAGGTCAACACATCAATG-3' |
| FL1112 | 5'-CTCTATTCCTTTGCCCTCG-3' |
| FL2450 | 5’-ATGATCCGCTCAACACAAATAG -3’ |
| FL2451 | 5’-CTGTTGCTTCGTCCAAATTCATCgatatcGATTTGGTTGTTGCTGAGTTGG -3’ |
| FL2452 | 5’-CCAACTCAGCAACAACCAAATCgatatcGATGAATTTGGACGAAGCAACAG -3’ |
| FL2453 | 5’- CAGGAACATCTCGAATGGTATC-3’ |
| FL2580 | 5’-ATGATCCGCTCAACACAAATAG-3’ |
| FL2481 | 5’-TCAAAAGAAACGCCACCAT-3’ |
| FL2701 | 5’-CTAGCTCGACGTCGCCGCACCT-3’ |
| FL3614 | 5’-AGTGCATATCTGCCCTTCTCTC-3’ |
| FL3615 | 5’-GAAATTCAACTATGCCCCTCTG-3’ |
| FL4348 | 5’-AGGTGCAGAACGGCGACTTCGAT-3’ |
| FL4394 | 5’-ACATGGACACCACCCAGAAC-3’ |
| FL4395 | 5’-CATGCTTCCCTCGAGACCAC-3’ |
| FL4396 | 5’-ATCAAGGCCGATGATGCTAC-3’ |
| FL4397 | 5’-ATGTGCTACCCAGACCCTTG-3’ |
| FL4779 | 5’-GTCACAGCGTGACGACAAGT-3’ |
| FL4780 | 5’-GTCCTCCTGTCGCTTCAGAC-3’ |
| FL4781 | 5’-TACTTTGACGACGCCATGAC-3’ |
| FL4782 | 5’-CCCGACGGTATGTGCTAAGT-3’ |
| FL4783 | 5’-GCGTCAACAACATCAACACC-3’ |
| FL4784 | 5’-AATCGGCTCTTGAGCTTACC-3’ |
| FL4793 | 5’-GAAACGATGCAGGTTTGTCC-3’ |
| FL4794 | 5’-CATATGCATGCACCAACACC-3’ |
| FL4795 | 5’-GGAGTACTGGCTCTCCATCG-3’ |
| FL4796 | 5’-TTGGTCTGCATGTTGTTGGT-3’ |
| FL4803 | 5’-TCCCTGGACTGCTCAAGTCT-3’ |
| FL4804 | 5’-GCTGTCAAGAGGACGGTAGC-3’ |
| FL4789 | 5’-CTTCCAGTACGAGGGTGCT-3’ |
| FL4790 | 5’-TGTTGACGGTCCAGTAGACG-3’ |
| FL4801 | 5’-ACCAACAACCTCACCAGGAG-3’ |
| FL4802 | 5’-GCTGGACCGTTGATGACGAT-3’ |
| FL4799 | 5’-CCACGAGCTCAACTTTGGAT-3’ |
| FL4800 | 5’-GGACGGTGACAAGCATCTCT-3’ |
| FL4787 | 5’-CTGCTGCTCAACGAGAAGTG-3’ |
| FL4788 | 5’-ACACCGAGCTCAAACAGCTT-3’ |
| FL4806 | 5’-ACAACGGTCGCCTTTTACAC-3’ |
| FL4807 | 5’-GTGTTGGTGTTGGCCTTTTC-3’ |
