# Supplementary material for: R-SNARE Homolog MoSec22 Is Required for Conidiogenesis, Cell Wall Integrity, and Pathogenesis of Magnaporthe oryzae
Source: PLoS One. 2010 Oct 6;5(10):e13193. doi: 10.1371/journal.pone.0013193 (PMC2950850; doi:10.1371/journal.pone.0013193)
Supplement: Table S2 — The ΔMosec22 mutant displays increased sensitivity to cell wall perturbing agents. (0.03 MB DOC) [file pone.0013193.s002.doc]

Table S2. The ∆*Mosec22* mutant displays increased sensitivity to cell wall perturbing agents ^a^.

| Growth medium | Growth-inhibiting rate^b^ (%) P>0.01 | | | |
| --- | --- | --- | --- | --- |
|  | Guy11 | *∆Mgsec22-#1* | *∆Mgsec22-#2* | *∆Mgsec22R* |
| CM | 0 | 0 | 0 | 0 |
| +200 μg/ml CFW | 0.27±0.03a ^c^ | 0.1±0.03b | 0.14±0.01b | 0.26±0.04a |
| +0.01%SDS | 0.39±0.02a | 0.23±0.01b | 0.23±0.04b | 0.41±0.05a |
| +200 μg/ml CR | 0.16±0.02a | 0.09b | 0.03±0.03c | 0.14±0.01ab |

^a^  The strains were incubated on solid CM medium supplemented with CFW (200 μg/ml), SDS (0.01%), and CR (400 μg/ml) at 28°C for 6 days. Values are the mean ± SD from at least three independent experiments, each with three duplicates.

^b^ Growth-inhibiting rate = [(Diameter on complete medium – Diameter on completer medium with stress)/ Diameter on complete medium] /100.

^c^ Different letters in each data line (for example: Guy11 v.s. *∆Mgsec22-#1*, *∆Mgsec22-#1* v.s. *∆Mgsec22-#2*, Guy11 v.s. *∆Mgsec22-#2*, and Guy11 v.s. *∆Mgsec22R* ) indicate significant differences at P<0.01.
