# Supplementary material for: R-SNARE Homolog MoSec22 Is Required for Conidiogenesis, Cell Wall Integrity, and Pathogenesis of Magnaporthe oryzae
Source: PLoS One. 2010 Oct 6;5(10):e13193. doi: 10.1371/journal.pone.0013193 (PMC2950850; doi:10.1371/journal.pone.0013193)
Supplement: Figure S1 — FM4-64 staining reveals that the ΔMosec22 mutant is defective in endocytosis. (3.11 MB PPT) [file pone.0013193.s003.ppt]

## Slide 1
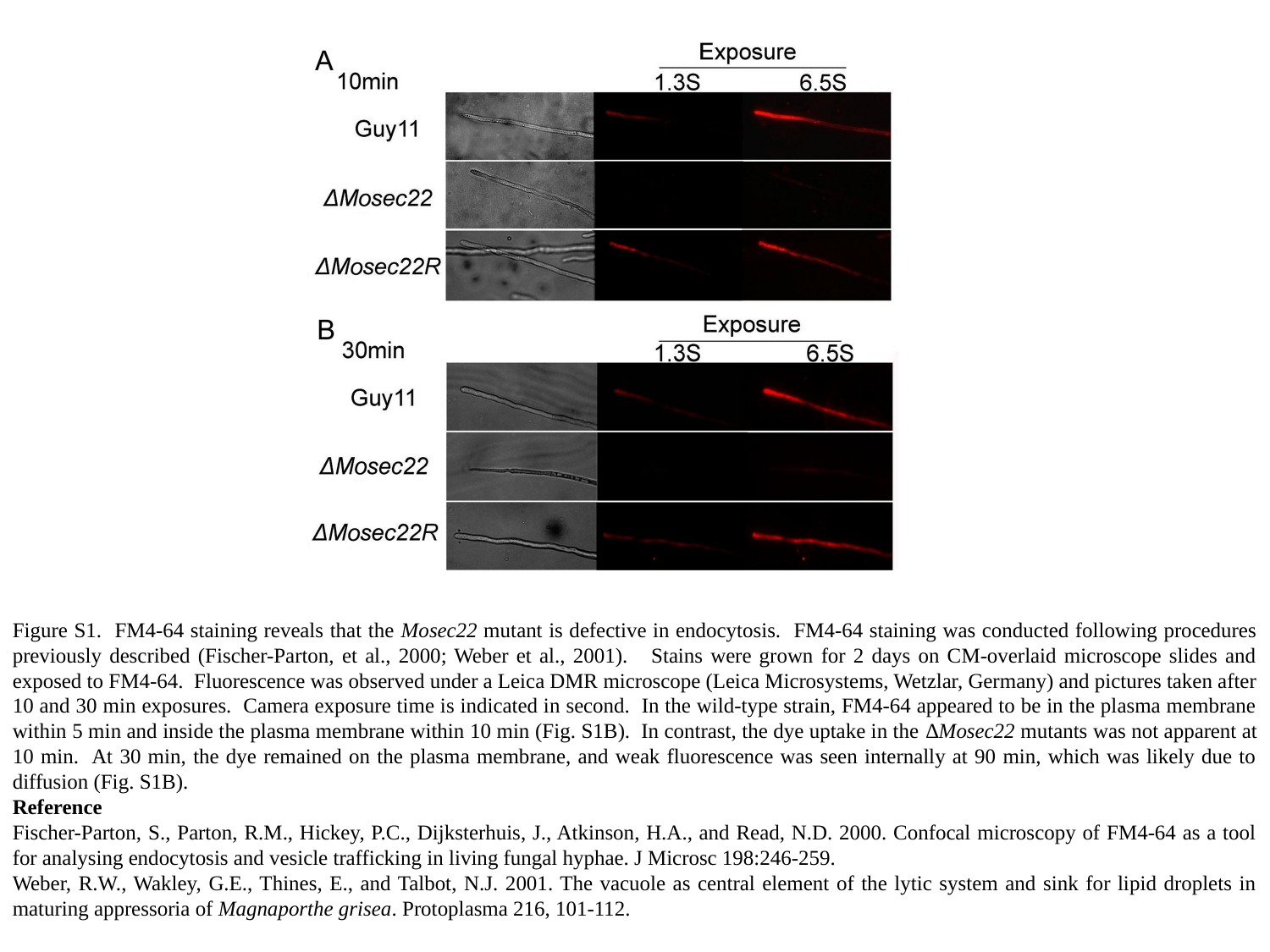

Figure S1. FM4-64 staining reveals that the Mosec22 mutant is defective in endocytosis. FM4-64 staining was conducted following procedures previously described (Fischer-Parton, et al., 2000; Weber et al., 2001). Stains were grown for 2 days on CM-overlaid microscope slides and exposed to FM4-64. Fluorescence was observed under a Leica DMR microscope (Leica Microsystems, Wetzlar, Germany) and pictures taken after 10 and 30 min exposures. Camera exposure time is indicated in second. In the wild-type strain, FM4-64 appeared to be in the plasma membrane within 5 min and inside the plasma membrane within 10 min (Fig. S1B). In contrast, the dye uptake in the ∆Mosec22 mutants was not apparent at 10 min. At 30 min, the dye remained on the plasma membrane, and weak fluorescence was seen internally at 90 min, which was likely due to diffusion (Fig. S1B).
Reference
Fischer-Parton, S., Parton, R.M., Hickey, P.C., Dijksterhuis, J., Atkinson, H.A., and Read, N.D. 2000. Confocal microscopy of FM4-64 as a tool for analysing endocytosis and vesicle trafficking in living fungal hyphae. J Microsc 198:246-259.
Weber, R.W., Wakley, G.E., Thines, E., and Talbot, N.J. 2001. The vacuole as central element of the lytic system and sink for lipid droplets in maturing appressoria of Magnaporthe grisea. Protoplasma 216, 101-112.
